# Visualizing histopathologic deep learning classification and anomaly detection using nonlinear feature space dimensionality reduction

**DOI:** 10.1186/s12859-018-2184-4

**Published:** 2018-05-16

**Authors:** Kevin Faust, Quin Xie, Dominick Han, Kartikay Goyle, Zoya Volynskaya, Ugljesa Djuric, Phedias Diamandis

**Affiliations:** 1 Department of Computer Science, University of Toronto, 40 St. George Street, Toronto, ON M5S 2E4 Canada; 2 Department of Laboratory Medicine and Pathobiology, University of Toronto, Toronto, ON M5S 1A8 Canada; 3 The Edward S. Rogers Sr. Department of Electrical & Computer Engineering, University of Toronto, Toronto, ON Canada; 4 Laboratory Medicine Program, Department of Pathology, University Health Network, 200 Elizabeth Street, Toronto, ON M5G 2C4 Canada; 5 Princess Margaret Cancer Centre, MacFeeters Hamilton Centre for Neuro-Oncology Research, 101 College Street, Toronto, ON M5G 1L7 Canada

**Keywords:** Digital pathology, Deep learning, Convolutional neural networks, t-SNE, Diagnostics, Neuropathology, Cancer, Glioblastoma, Artificial intelligence, Machine learning

## Abstract

**Background:**

There is growing interest in utilizing artificial intelligence, and particularly deep learning, for computer vision in histopathology. While accumulating studies highlight expert-level performance of convolutional neural networks (CNNs) on focused classification tasks, most studies rely on probability distribution scores with empirically defined cutoff values based on *post-hoc* analysis. More generalizable tools that allow humans to visualize histology-based deep learning inferences and decision making are scarce.

**Results:**

Here, we leverage t-distributed Stochastic Neighbor Embedding (t-SNE) to reduce dimensionality and depict how CNNs organize histomorphologic information. Unique to our workflow, we develop a quantitative and transparent approach to visualizing classification decisions prior to softmax compression. By discretizing the relationships between classes on the t-SNE plot, we show we can super-impose randomly sampled regions of test images and use their distribution to render statistically-driven classifications. Therefore, in addition to providing intuitive outputs for human review, this visual approach can carry out automated and objective multi-class classifications similar to more traditional and less-transparent categorical probability distribution scores. Importantly, this novel classification approach is driven by *a priori* statistically defined cutoffs. It therefore serves as a generalizable classification and anomaly detection tool less reliant on *post-hoc* tuning.

**Conclusion:**

Routine incorporation of this convenient approach for quantitative visualization and error reduction in histopathology aims to accelerate early adoption of CNNs into generalized real-world applications where unanticipated and previously untrained classes are often encountered.

**Electronic supplementary material:**

The online version of this article (10.1186/s12859-018-2184-4) contains supplementary material, which is available to authorized users.

## Background

### Need for visualization and outlier detection tools in histopathologic deep learning models

The personalization of medical care has substantially increased the diagnostic demands, workload, and subspecialty requirements in pathology. As a result, there is an emerging interest in leveraging artificial intelligence (AI), and especially deep convolutional neural networks (CNNs), to augment the diagnostic capabilities of pathologists [1–3]. Numerous studies have already shown expert-level performance of CNNs [4–6] in a diverse array of histopathologic classification tasks [7–9]. However, bias for narrow, often binary readouts limit application for more generalizable classification workflows involving multiple output and unanticipated classes. Most CNN classification approaches so far have relied on empirically generated probability distribution scores that are described to lack transparency (e.g. “black box”) and generalizability. When using CNNs optimized for only two classes, high probability scores (approaching a value of 1.0), signify a strong likelihood of a given diagnosis (high specificity). Using such high cutoff values, however, can compromise sensitivity. Similarly, lower probability score cutoffs for a specific class, although improve sensitivity, risk misclassification. For binary and highly focused tasks, “cutoff” values can be empirically optimized through receiver operator characteristic (ROC) curves generated on *post-hoc* analysis. Challenges to this binary approach arise when multiple output classes are considered. Similarly, in practical “real-world” scenarios, unanticipated technical artifacts and previously untrained or validated classes can compromise extrapolation of these chosen cutoff values. Recent attempts in colon cancer [10] highlight these challenges. While accuracy rates for distinguishing two classes reached 98.0%, generalizing classification to five different colon cancer subtypes (conventional, mucinous, serrated, papillary and cribriform comedo-type adenocarcinoma) and normal tissue reduced accuracy to 87.5% [10]. In the later multi-class example, probability score cutoffs become exceedingly more context-specific and highly dependent on the relative distribution of scores amongst the available classes. Although the performance of these complex and generalized tasks can be theoretically resolved with massive and comprehensive training examples, development of transparent approaches to visualize and efficiently detect anomalies offers a more immediate and global solution to accelerate adoption of CNNs into practical everyday use.

Here we show how nonlinear dimensionality reduction using t-distributed stochastic neighbor embedding (t-SNE) [11–14] can provide informative planar representations of high dimensional histologic data structures of CNNs prior to softmax transformation. As relationships between pairs (local) and clusters (global) of images are organized in t-SNE space using distance metrics, how a computer perceives intra- and inter-class morphologic similarities can be easily visualized and inferred. Furthermore, we demonstrate how t-SNE plots can be leveraged to visualize CNN-driven histological classifications. Importantly, unlike the continuous probability distribution scores that are divided only amongst the defined classes as a continuous sum, this approach allows images to be categorized in both learned and undefined classes within the t-SNE plot. We show that this discretized information can be leveraged to provide an innate and statistically driven approach for classification and outlier detection that is less dependent on *post-hoc* ROC curve-based tuning. Moreover, despite being derived from the same training data, we show a composite approach to classification (t-SNE + probability score) can serve to further improve the performance in novel settings. These novel enhancements serve as generalizable tools to improve adoption of more diverse and unsupervised classification tasks in diagnostic pathology.

### Surgical neuropathology as a model for complex histopathological decision making

Diagnostic neuropathology, the branch of pathology focused on the microscopic examination of neurosurgical specimens, is a challenging skill requiring multiple years of training for humans to reach adequate proficiency. Firstly, because of their location, neuropathological specimens are usually small, often intermixed with non-lesional tissue (e.g. normal brain, blood, surgical cloth) and represent only a small sample of the overall disease. Classification is further challenged by the brain’s multiple anatomical structures (e.g. white matter, gray matter and cerebellar cortex) that each have distinct morphology. To the non-subspecialized pathologist, even normal tissues can be sometimes be mistaken as an abnormality. Once the lesion is correctly located, the pathologist must then determine if the abnormality represents a neoplastic or non-neoplastic lesion. The most common primary brain neoplasms encountered include gliomas (tumors of resident brain cells), meningiomas (tumors arising from the brain’s leptomeningeal covering), and schwannomas (tumors arising from the nerve’s Schwann cells). It is also very common for tumors originating outside the brain to form deposits within the nervous system (metastases). Differentiating these tumors is an important task as some can be managed effectively with surgery alone, while others require additional chemo- and radiation therapy. Although less common, it is essential for a pathologist to rule out the presence of a lymphoma, a form of blood cancer in which patients do not benefit from aggressive surgery and should be triaged to early initiation of chemotherapy. To reach one of these biologically distinct diagnoses, a pathologist first uses microscopic information from tissue stained with hematoxylin and eosin (H&E). This staining technique accentuates the resolution of distinctive cellular patterns that are characteristic of the different diseases. Gliomas and lymphomas, for example, tend to be “discohesive” and grow as individual cells within the brain tissue. Meningiomas and metastasis on the other hand, tend to grow as cohesive collections and clusters of cells. Meningiomas can also sometimes resemble schwannomas when they take on a more spindled arrangement. While the integration of multiple features usually allows a pathologist to arrive at a specific diagnosis, oftentimes, the partially overlapping patterns can make this a challenging task. In many cases where a specific diagnosis cannot be reached, the pathologist can devise a “differential diagnosis”. This short list of diagnostic possibilities can then be further differentiated using more definitive molecular techniques (e.g. sequencing, immunohistochemistry). Sometimes, for rare and very atypical cases, pathologists can initially label a case as “undefined” and perform a broader workup to reach a final diagnosis. While these five tumor types discussed represent the majority of cases typically encountered in diagnostic practice (~ 75–80%), there are in fact over 100 different brains tumor subtypes and many more non-neoplastic diseases that need to be considered [15, 16]. Some of these subtypes/variants are exceedingly rare with pathologists encountering a single case once every decade (or lifetime). Similarly, new diseases (e.g. Zika encephalitis) continually arise. To the unsuspecting pathologist, these rare and evolving cases, that together amass to a relatively common diagnostic group, often lead to misclassifications. The ability to identify these rare and anomalous cases and help triage appropriate molecular testing is a highly valuable and cost-effective skill. This logical and “graded” approach to classification (e.g. diagnosis, differential, undefined) thus provides an attractive blueprint to designing practical “real-world” decision support tools for pathologists. In addition to confirming diagnoses of common tumor types, machine classifiers, like humans, should be able to signal these different degrees of uncertainty, especially for rare and novel classes that may not have been encountered during model training.

## Methods

### Development of an image training set

**Fig. 1:**
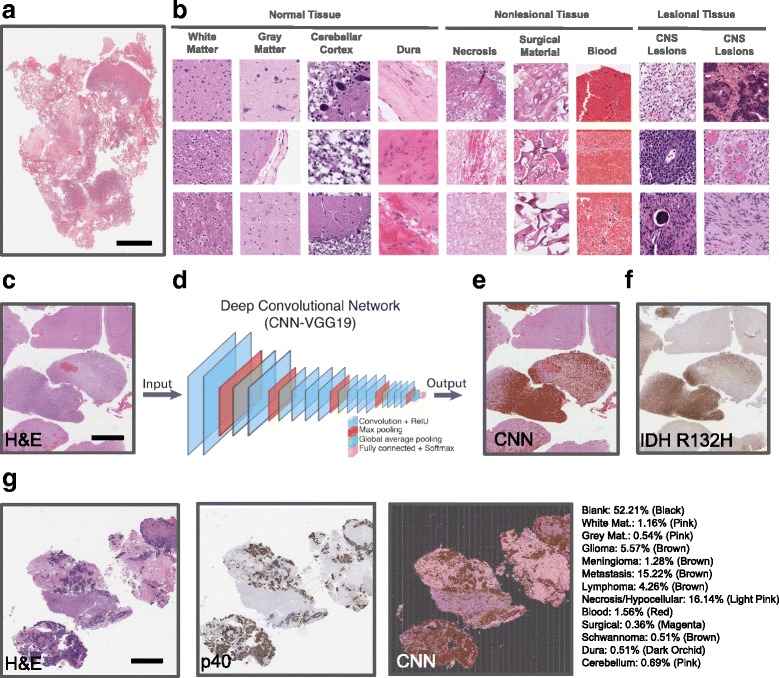
Development of a multi-class classification model of CNS tissue using CNNs. **a** H&E-stained WSI of a glioblastoma containing a heterogeneous mixture of tumor, necrosis, brain tissue, blood and surgical material. Black scale bar represents 4 mm. **b** Examples of image tiles for the 13 classes used for CNN training are shown. Images have been magnified to ~ 250 μm^2^ to highlight key diagnostic features. **c-e** WSI-level annotations are carried through automated tiling and classification of 1024 × 1024 pixel image patches using our trained CNN. Class activation maps (CAMs) are generated by reassembly of classified tiles to provide a global overview of lesion localization (brown). Black scale bar represents 2 mm. **f** Immunohistochemistry for IDH1-R132H shows the associated “ground truth” for this glioma. **g** H&E section of a metastatic carcinoma (left panel), associated ground truth (middle panel, p40 immunostaining) and the lesional coordinates (brown) predicted by the CNN. The aggregate probability scores generated by the final softmax function allows for global estimates of the various tissue types found on each WSI. Black scale bar represents 3 mm

Slides from our neuropathology service were digitized into whole slide images (WSI) on the Aperio AT2 whole slide scanner at an apparent magnification of 20× and a compression quality of 0.70. We reviewed a collection of 122 slides to generate a growing class list of common tissue types and lesions encountered in our practice (Additional file 1: Table S1, Fig. 1a-b). For each tissue class, based on availability, we manually generated a collection of 368–18,948 images patches (dimensions: 1024 × 1024 pixels). For some classes, such as surgical material, only a small number of high quality tiles could be generated. For other more abundant classes, we limited training tile numbers to 7000 to avoid skewed representation of specific groups that could affect overall training and performance. For this study, we focused our lesion categories on the most common and important nervous system neoplasms (Additional file 1: Table S1): gliomas, metastatic carcinomas, meningiomas, lymphomas, and schwannomas. We chose a tile size (image patch) of 1024 × 1024 pixels (0.504 μm per pixel, 516 μm^2^) to carry out training and classification, a tile size over 10 times larger than most other approaches [2]. We found this larger size excels at complex classification tasks by providing multiple levels of morphologic detail (single cell-level and overall tumor structure) without significantly affecting computation times. We found larger tile sizes significantly impede training efficiency without improving accuracy. Similarly, many of the distinguishing architectural features of tumors where not appreciable at smaller patch sizes and compromised performance. All tile annotations were carried out by board-certified pathologists. Only the diagnosis relating to the lesional tissue on each slide was extracted from the medical records and all images were otherwise anonymized. The University Health Network Research Ethics Board (REB) approved our study.

Our CNN was designed with 2 specific objectives in mind. Firstly, we chose a collection of training cases that included the most common tumor and tissue elements found in routine practice. We felt this would help develop a relatively well-performing classifier that encompassed most of the expected classes it would encounter. As the main objective of our study was to develop a workflow that could handle the different degrees of uncertainty described above (diagnosis, differential diagnosis, undefined), we did not include an authoritative collection of additional uncommon tumor types. This more focused classifier would allow us to encounter a sufficient number of “novel” and untrained classes in our unselected group of test cases. By including lesions that comprise about 75–80% of cases typical seen in our validation cohort, we expected 20–25% of randomly selected test cases to collectively represent an aggregated class of “outlier cases”. Our goal was to see if we could develop an approach to efficiently flag this group of cases as “anomalous” (differential diagnosis or undefined) rather than erroneously misclassifying them.

### Convolutional neural network (CNN) optimization

**Fig. 2:**
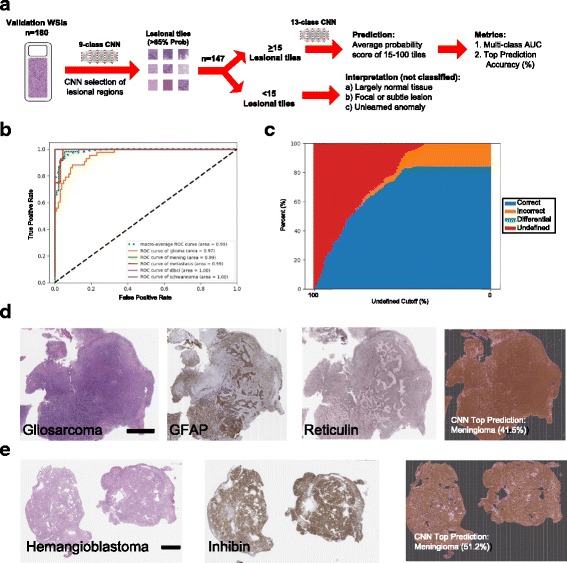
Probability score-based classification workflow and performance. **a** Automated lesion segmentation and classification workflow for 180 prospective and randomly selected WSIs of cerebral lesions. Only image tiles with a lesional probability score of > 85% were used for class predictions. To reduce noise, classification was only carried out on WSIs with > 15 lesional tiles (*n* = 147). The majority of unclassified WSIs (*n* = 33) represented non-neoplastic processes (e.g. epidermoid cysts, hemorrhage, normal brain tissue). **b** Multi-class ROC curves were empirically generated by deriving the sensitivity (fraction of detected true positives) and specificity (fraction of detected true negatives) at different probability score distribution thresholds. The displayed AUC is a measure of performance with a minimum value of 0.50 (random predictions) and 1.0 (all correct predictions). **c** Relationship of the accuracy of the top classification output at different minimum probability score cutoffs. If this cutoff value is not reach, the case is deemed “undefined” and not included in the scoring. This empirical *post-hoc* analysis highlights a specific threshold where the error rate substantially rises. **d** A H&E-stained validation WSI of a gliosarcoma (glioma subtype), confirmatory special stains and the CAM showing the top CNN probability score-based prediction. In this study, we define these misclassification between lesion types as Type B errors. Black scale bar represents 4 mm. **e** An example of an erroneously classified tumor type (hemangioblastoma) that was not included in this 13-class model (“Type C error”). Black scale bar represents 3 mm

To make our workflow more generalizable to others in the field, we specifically chose to use a pre-trained and widely available CNN rather than developing our own CNN architecture. Specifically, we took advantage of the pre-trained VGG19 CNN [17] for lesion segmentation and classification. VGG19 is a popular 19-layer neural network comprising of repetitive convolutional layer blocks previously trained on over 1.2 million images in the ImageNet database. This network architecture, similar to other CNNs, outperforms conventional machine learning algorithms at computer vision tasks such as classifying images containing 1000 common object classes. Importantly, VGG19 has a strong generalizability with the ability to transfer learned image features (e.g. edges, lines, round shapes, etc.) to other image classification tasks through fine-tuning with additional task-specific images. To carry out this process, we loaded VGG19 into Keras with a Tensorflow backend and retrained the final 2 convolutional layer blocks of the network using our collection of annotated pathology images. While there are multiple training approaches, focusing on the final layers substantially reduces training times and effectively tunes and optimizes CNNs for catered pattern recognition tasks including pathology [18]. Specifically, this VGG19 CNN was retrained using 8 “non-lesional” object classes commonly found on neuropathology tissue slides: hemorrhage, surgical material, dura, necrosis, blank slide space and normal cortical gray, white and cerebellar brain tissue. In addition to this, image tiles of the most common nervous system tumor types (gliomas, meningiomas, schwannomas, metastases and lymphomas) were included either separately (13 class model) or as a single common lesion class (9 class model). We used the 9-class model to separate “lesional” regions from normal tissue and then used the 13-class model to classify the identified regions. These respective training image sets were used to retrain and optimize the VGG19 neural network to act as a lesion segmentation and classification tool. Specifically, training images were partitioned into training and validation set in a 85:15 ratio and optimized through back-propagation over a series of 300 potential epochs (Additional file 2: Figure S1). The best performing model was selected for further independent testing. Testing, highlighted in Figs. 1 and 2, was carried out by averaging the resulting probability distribution scores generated by the CNN’s final softmax function. All steps, including random tile selection, training, and validation were automated using the Python programming environment and powered by an NVIDIA Titan Xp graphical processing unit (GPU).

### Development of a multi-class CNN-based histologic classifier

To develop a baseline level of performance for multi-class histopathologic decision making in a practical (“generalized”) environment, we trained the widely available VGG19 CNN on 13 common tissue and lesion classes encountered in surgical specimens of the central nervous system (CNS) (Fig. 1a-b). Our training set was comprised of a local, randomly selected cohort of 47,531 pathologist-annotated hematoxylin and eosin (H&E)-stained image patches taken from a larger pool of 84,503 images (Additional file 1: Table S1, training set can be downloaded from https://zenodo.org/). We used these images to retrain the final layers of the VGG19 CNN (Fig. 1d). During this process of transfer learning, our additional images served to help fine-tune and customize previously learning patterns and CNN weights towards the histopathologic features found within our 13 tissue classes. Our model reached a validation accuracy of 94.8% after 300 epochs (Additional file 2: Figure S1). Compared to more focused approaches that train CNNs with 2–3 tissue classes, our 13-class model demonstrates that deep neural networks can be effectively trained to differentiate between a large number of histological classes.

### t-distributed stochastic neighbour embedding (t-SNE) visualization and classification

t-distributed Stochastic Neighbour Embedding (t-SNE) [11] was used to help visualize the high-dimensional relationships of the 13 learned classes on a two dimensional plane. Specifically, we plotted a random selection of approximately 350–600 training image tiles for each class. Further optimization was carried out to automate removal of potentially misclassified training images or tiles containing features of multiple classes. To remove these potentially anomalous points, we compared each point on the *t*-SNE plot to its nearest 300 neighbors to determine if points substantially deviated from their labeled class cluster. This provided a refined visual plot highlighting the learning relationship of representative tiles and classes to one another.

Specifically, for this study, we wanted to use this initial map to develop a visual classification and anomaly detection tool. Towards this, we used the spatial distribution of up to 100 representative tiles generated from each test/validation image to carry out classification at the tile and WSI level. For this, we leverage the generated t-SNE to visualize where new image tiles lie within the two-dimensional plot. This discretized approach allowed determination of what cluster (class) each testing tile belonged to, or whether it represented an undefined “outlier” image. Using the tile images that were fed into the earlier t-SNE, we add the new tiles and regenerated the t-SNE for each WSI. Although the resulting t-SNE is slightly altered from the original with the addition of new data, the spatial structure and clustering of classes remains largely preserved. To classify new tile points, we first assess if each image tile represents an outlier. This is achieved by looking at its closest 25 neighboring points to determine if at least 85% of them fall into a single class. If the condition is satisfied, the tile is discretized (categorized) to represent this class for classification; otherwise it is labeled as an outlier/anomalous data point. We felt this relatively conservative approach would allow classification to only rely on information from the slide that most closely resembles the previously trained examples.

**Fig. 3:**
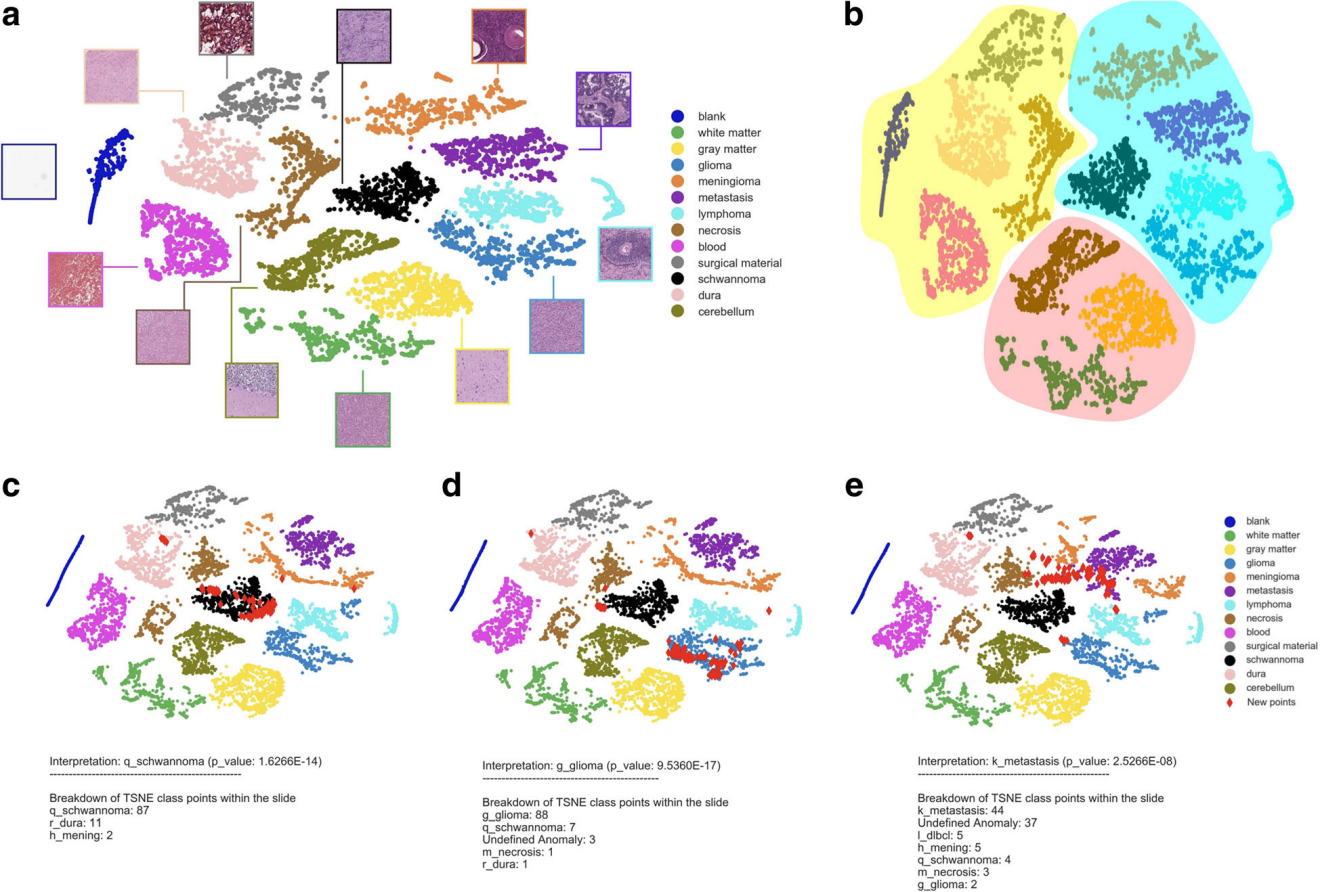
Visualization of CNN-based histological data structure and classification using t-SNE. **a** t-SNE plot showing the planar representation of the internal high-dimensional organization of the 13 trained tissue classes within the CNN’s final hidden layer. 350–600 training tiles from each class are plotted so that each point within the t-SNE represents a 1024 × 1024 pixel training image. Tiles belonging to each class are labeled with a unique colour for convenience. Insets show representative images from each cluster/class. **b** Dimensionality reduction techniques (like t-SNE) position data so that points close together represents images the CNN perceives as have a similar pattern. This plot therefore allows visualization of what classes the computer perceives to be closely related. Learned features appear to qualitatively organize in a biology-inspired manner similar to the framework shown in Fig. 1b. In addition to anuclear (yellow region), normal (red region) and lesional (blue region) tissue regions, there is an additional trend towards cohesive lesions (meningioma and metastasis) being arranged close together as one moves upward within the large blue cluster. Understanding such configurations could provide more transparency into computer-driven learning of medical images. **c**-**e** Examples of t-SNE-based visualization and classification of test WSIs. For each prediction, we overlay 100 images patches extracted from testing images (represented by the red diamonds) to carry out classification. A k-nearest neighbor approach is used to assign individual tiles to clusters or undefined regions. In addition to qualitative visual predictions, the distribution of testing tiles (χ^2^ test) allows for quantitative statistically driven classification scores. Clinicopathological classes: schwannoma (**c**), glioma (**d**) and metastasis (**e**)

For t-SNE classification on the WSI-level, up to 100 random lesional tiles extracted from each test image were plotted on the CNN’s t-SNE map. As slides may contain a few “background” non-lesional tissue and artefacts that may focally resemble pathology, we did not carry out classification on a slide if less than 15 “lesional” tiles were generated. Instead, our workflow flags these slides and provides a handful of lesional tiles for manual inspection by the pathologist (See Additional file 2: Figure S2). Otherwise, using the above approach, we determined the classes of each image tile and exported them to a contingency table to statistically analyze their distribution. We use the distribution of these 15–100 tiles to carry out an iterative *χ*^2^ testing process, where the class with the fewest tiles is systematically removed and the remaining distribution is retested. This process continues until the *χ*^2^ score (*p*-value) is no longer significant (*p* ≥ 0.01). This process either leads to a single diagnosis (Fig. 3) or a list of classes (“differential diagnosis”, Additional file 2: Figure S3) where the distribution of tiles is not significantly different when compared to a random, equally partitioned distribution amongst the remaining cases. If a statistically significant distribution of plotted tiles (*χ*^2^ test, *p* < 0.01) are labeled as “undefined/outliers” on the first iteration, the WSI is deemed to contain too many novel/anomalous features to render a confident diagnosis. These slides are thus classified as “undefined”. This p-value can be tuned *a priori* to the tolerable α error. Given the size of our testing set (180 slides), we chose a cutoff score of *p* < 0.01. As a comparative analysis, we carried out the same classification approach using principle component analysis, another commonly used dimensionality reduction and visualization tool (Fig. 6). Similarly, to highlight the effect of using low testing tiles thresholds for classification, we reanalyzed out testing cohort with a minimum tile cutoff of 5 instead of 15 (Additional file 2: Figure S4).

### Performance testing

Performance of the same CNN was evaluated in a number of ways on a prospective, randomly selected set of cases comprising of 180 WSIs (Table 1). To improve generalizability, we chose not to bias test case selection or to focus on a specific anomaly. To maximize inter- and intra-case diversity, when available, we included up to 5 slides of any single case. This resulted in a testing set with both prevalent and less common lesion types (representative WSI testing images can be downloaded from www.zenodo.com). Similar to the generation of the training set, this validation set was restricted to cases in which consensus was reached by at least 3 board-certified pathologists with extensive neuropathology training (years of practice: 2, 15, 22, 31). The rendered diagnosis was used as the “integrated clinicopathologic diagnosis” for performance testing. All cases and diagnoses also benefited from confirmatory immunostaining (Figs. 1 and 2) and/or corroborating clinical correlates (e.g. location, radiological impression). We felt this integrated approach would help reduce subjective interpretive errors and establish a well-approximated “ground truth” [19, 20].

**Table 1:** Distribution of WSI in validation cohort

Diagnosis	Unique Slides	Unique Cases	> 15 Lesion Tiles)	Misclassified by Prediction score	Misclassified by t-SNE	Misclassified by both
Trained Classes						
Glioma	47	15 12 Glioblastoma, WHO IV, IDH-wt 1 Anaplastic Astrocytoma, WHO III 1 Anaplastic Oligo, WHO III 1 Gliosarcoma, WHO IV	43/47	8/43	4/25	2/23
Meningioma	57	18 15 Meningioma, WHO I (Meningothelial, Angiomatous, Fibrous, Transitional) 3 Atypical Meningioma, WHO II	55/57	1/55	1/47	1/47
Schwannoma	23	7 Conventional Type, WHO I	23/23	0/23	0/22	0/22
Metastasis	12	7 3 Lung Adenocarcinoma 1 Lung Squamous Cell Carcinoma 1 Breast Adenocarcinoma 1 Esophageal Adenocarcinoma 1 Squamous Cell Carcinoma, NYD	12/12	3/12	0/9	0/9
Lymphoma	2	1 Diffuse Large B-Cell Lymphoma	2/2	0/2	0/1	0/1
Hematoma	15	3	3/15	2/3	1/1	N/A
Brain Tissue	3	2	1/3	1/1	N/A	N/A
Dura	1	1	1/1	1/1	0/1	N/A
Novel Classes						
Hemangioblastoma	5	2	5/5	5/5	N/A	N/A
Radiation necrosis	3	1	2/3	2/2	N/A	N/A
Focal Cortical Dysplasia	5	1	0	N/A	N/A	N/A
Cartilaginous Material	5	2	0	N/A	N/A	N/A
Epidermoid Cyst	2	1	0	N/A	N/A	N/A
Total: 13 Class	180	61	82	23	6	3

WSI were randomly selected from prospective cases from our local surgical neuropathology service. All cases selected had diagnostic consensus amongst 3 board certified neuropathologists and had confirmatory immunohistochemical staining patterns. Up to 5 slides of the same cases were used when available

**Fig. 4:**
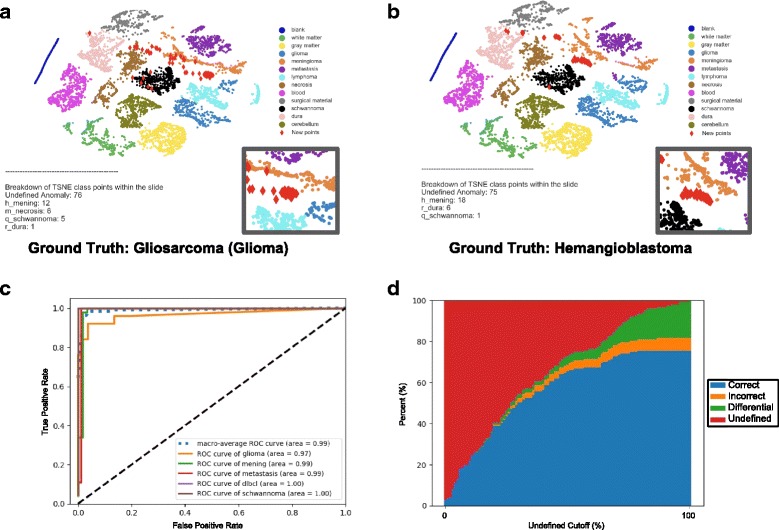
Detection and visualization of histopathologic outliers using t-SNE. **a-b** t-SNE-based WSI visualization and classification of a gliosarcoma (rare glioma subtype) (**a**) and a hemangioblastoma (**b**). Unlike previous examples, these lesions represent patterns and tumor types never previously encountered by the CNN. Localization of the vast majority of lesional tiles within the unoccupied space allows confident visual and statistical classification as an “outlier” without the need for a reference ROC curve. Insets (lower right) magnify the localization of tiles in unoccupied space. These examples demonstrate how the properties of the t-SNE plot can be leveraged to detect erroneous classification of novel/challenging cases. **c**. ROC performance summary on the same set of test WSIs used in Fig. 2. Classification using t-SNE tile distributions yields a similar performance (AUC) metric to the probability score-based approach. **d** relationship of t-SNE accuracy at different defined “outlier” cutoffs for comparison. Although more conservative in WSI classification, this t-SNE approach shows a more uniform performance (orange; error rate) across different “cutoff scores”. This distinct feature improves its generalizability when cut-off values cannot be reliably or empirically estimated

For each WSI, a diagnosis was generated using the probability distribution scores of the CNN’s final softmax output layer or the tile distribution overlaid on the t-SNE or PCA plot of the CNN’s final hidden layer. We also generated composite-based predictions (combined and hybrid approaches) *post-hoc* to understand if errors between the different approaches overlapped. To test the performance of these different classifiers, the various multi-class metrics were used to generate a ROC curve and calculate the occupied areas under the ROC curve (AUC). While some approaches collapse multiple outputs of a multi-class classifier into a binary readout for ROC and AUC analysis, we found that the distribution of probabilities and tiles amongst the classes substantially influenced the confidence of a specific diagnosis. We thus opted to use the distribution of probability scores and tiles to approximate the multiclass ROC (mROC) as previously described [21]. In addition to AUC analysis, we also assessed the performance of each approach by computing the proportion of cases classified correctly or incorrectly (accuracy) compared to the consensus clinicopathologic diagnosis for each WSI. Throughout the text, these performance values are quoted without the use of specific cut-offs derived from the ROC curve. These and other *post-hoc* analyses are, however, provided as a reference (Figs. 2c & 4d). For simplicity, when comparing between different parameters and approaches, we use the class with the highest probability score to represent the diagnosis. For t-SNE performance testing, WSI images that were classified as “undefined” or as a “differential diagnosis” were not included in the testing as they were deemed outliers.

## Results

### Probability distribution score-based classification performance

We explored the baseline performance of this 13-class CNN on a prospective set of 180 randomly selected and digitized neuropathology whole slide images (WSIs) from our department (Fig. 2a). Given the expected frequencies of the 5 trained lesion types, this would provide a relatively large fraction of cases that the CNN would be able to correctly classify. At the same time, it would allow for a good proportion of untrained cases (~ 20%) to be encountered. Collectively, this later group would allow us to understand how novel and untrained histopathologic classes are handled by our CNN.

To visually monitor which regions of the WSI our CNN used for diagnosis, we systematically tiled WSIs into 1024 × 1024 pixel patches and overlay class activation maps (CAMs). These CAMs color code the tissues types and location, found within each tile. Reassembly of these tiles helped create fully annotated WSIs to qualitatively assess lesion segmentation performance. Comparison to expert pathologist-annotations and immunohistochemical staining showed strong concordance (Fig. 1c-g, Fig. 2d-e). This suggested that our 13 class CNN could efficiently differentiate lesion and non-lesion tissue classes for downstream analysis. We also averaged the confidence scores generated from each tile to provide a global estimate of the different tissue types detected by the CNN (Fig. 1g).

Clinically, a pathologist’s overall diagnosis is typically driven by the most abnormal tissue elements found within a slide. To steer classification (AI-based decisions) to these diagnostic (“lesional”) areas, we incorporated a more directed approach to classification testing (Fig. 2a). Rather than using the average of the WSI (e.g. Fig. 1g), we focused on image tiles that the CNN perceived to be enriched in lesional tissue (> 85% probability score) for averaging and classification. To avoid classification errors arising from focal artifacts, we further limited classification to WSIs in which at least 15 “lesional” tiles were identified (Additional file 2: Figure S2 & S4). Using this approach, 147 of the total 180 test slides met the threshold for classification by the CNN’s initial pass. As anticipated, the vast majority of the slides that were not classified by this approach comprised either of normal tissue or dramatically different pathologies that do not show any resemblance to trained classes (e.g. epidermoid cyst, Additional file 2: Figure S2). Using the distribution of prediction scores across the 13 classes for each WSI, this approach achieved a performance, as assessed by the areas under the multi-class receiver operator curve (AUC, mROC), of 0.99 (Fig. 2b). We also compared the accuracy of the class with the highest prediction score (“diagnosis”) to the integrated clinicopathologic diagnosis (Table 1). From the 147 slides examined, 84% were correctly classified (error:16%) by using the top ranked class type without knowledge of the optimal cut-off score (Fig. 2c). Three types of classification errors were identified. Misclassification of normal tissue for lesion (“Type A error”) was rare. This was likely due to the conservative pre-selection filter applied (> 15 lesional tiles of > 85% probability). This initial filter likely also helped flag some previously untrained lesions (e.g. epidermoid cysts) with distinct morphologic features. The error rate of the top class rose to 20% when we lowered this “lesional tile” cutoff for classification from 15 to 5 “lesional” tiles. (Additional file 2: Figure S4). The most notable Type A error identified was the CNN mistaking the normal, yet relatively cellular, cerebellar granular cell layer for a glioma. The true lesion in this specific WSI was a relatively small focus of metastatic carcinoma that was sub-optimally sampled due to the abundance of cellular cerebellar tissue. Such errors could likely be mitigated by more comprehensive sampling of normal cellular tissue types for training (Additional file 1: Table S1). A more common error type was misclassification between lesion types (“Type B error”). These largely represented misclassification of rare atypical variants of trained classes in our dataset (e.g. glioma vs gliosarcoma, Fig. 2d). In this example, the dominating spindled “sarcomatoid” component of the glioma was mistaken for meningioma; a tumor type that more often shows a similar morphology. Similarly, an atypical meningioma (WHO grade II) found in the test set, had prominent nucleoli and was not well represented in the initial training set of more benign meningioma images. This likely explained the misclassification as a metastasis. The third encountered error type (“Type C error”) was attributed to misclassification of novel and previously untrained tumor classes (e.g. hemangioblastoma, Fig. 2e). Type C errors in this validation set represented 5% of errors. The remaining misclassifications (11%) were largely attributed to the described “Type B” errors.

There are many approaches that can be used to address these different error types and improve performance. These include massive expansion of training images. Additional sampling of variants of existing classes (e.g. atypical meningioma) could potentially help find distinct and subtle differences between classes that are often misclassified. This could help reduce “Type B” errors (Fig. 2d). Similarly, incorporation of additional, previously untrained, classes can be incorporated into the CNN to reduce “Type C errors” (Fig. 2e). Another commonly used approach to increase specificity of an existing classifier is the use of *post-hoc* ROC-selected classification thresholds. While effective in their own right, these approaches poorly generalize beyond highly “controlled” tasks. While developing an alternative classification tool, we therefore chose to devise a more generalizable and *a priori* statistically-driven approach to anomaly detection and error reduction. Such an approach could offer more immediate solutions to help implement CNNs into more practical environments.

### Visualizing CNN data structure and classification decisions using dimensionality reduction

Most CNN-based histologic classification tasks commonly rely on probability distribution scores to categorize new image patches. Although convenient, averaging probability scores of image patches, especially when multiple classes exist, can introduce noise and reduce transparency of classifications. Moreover, optimization of classification thresholds is challenging when novel or atypical cases are often encountered in “real-world” settings.

Towards developing a more translucent and statistically driven approach to classifying cases in suboptimal settings, we take advantage of a complementary visualization tool to depict how histologic learning is organized within CNNs. For this, we chose to project representative training image tiles from each of the 13 tissue classes onto planar representations of the CNN’s higher-dimensional coordinates using t-SNE [11] (Fig. 3a**,** Additional file 3: Movie S1). Intriguingly, in addition to showing local organization of image tiles, this t-SNE plot also provided a more global two-dimensional arrangement of how the entire dataset is organized within the CNN. Qualitative inspection of the t-SNE plot shows an organizational framework within the CNN that mirrors understood biologic properties of the different tissue classes (Fig. 3b). For example, there is a prominent “cluster of clusters” (red circle) that arranges normal neural tissue types in close proximity to one another. This could represent the regular repeating pattern of these tissue types. This cluster appears to bisect the remaining tissue classes based on cellularity. This organizes hypocellular tissue classes on the left (yellow circle) and hypercellular lesional classes, forming a 3rd distinct cluster on the right (red circle). Further examination of the clusters suggests additional levels of a rational (and somewhat “humanoid”) organizational framework with discohesive lesions such as lymphoma and gliomas showing a close relationship. Notably, intrinsic brain tumors (gliomas) show the closest position to the included normal nervous system tissue elements (red cloud). Similarly, images of more cohesive neoplasms (e.g. metastases, meningiomas, schwannomas) cluster close together at the upper bound of the blue cloud on the t-SNE plot. This steady state representation map was generated in independent sampling and training experiments, suggesting convergence towards a stable learned global data structure for these included class types (Fig. 3c-e).


**Additional file 3: Movie S1.** Movie showing the 3D t-SNE representation of our trained CNN model. (MP4 2593 kb)


In addition to providing visual insights into CNN-based histologic learning, we investigated if t-SNE plots could provide more transparent decision-support outputs to humans when presented with new histological images. While this technique has been used by others to qualitatively visualize classifications and outliers [22], we wanted to develop a more automated, quantitative and statistically-driven approach to classification and anomaly detection. By overlaying representative lesional image tiles of new WSIs onto the 13-class t-SNE plot, we found that classifications could be made in a much more visual and intuitive manner (Fig. 3c-e). Similarly, since predictions of each tile can be discretized into single classes based on their proximity to neighboring training tiles (k-nearest neighbors), tile distributions can be statistically interrogated *a priori* for each case with less reliance on *post-hoc* ROC-generated cutoffs. Specifically, we used highly skewed class tile distributions (*χ*^2^ test, *p* < 0.01) to provide classifications (Fig. 3c-e). In a similar manner, less skewed test tile distributions in the observed class frequencies could be used to represent diagnostic “uncertainty” in the form of a “differential diagnosis” of the most populated classes (Additional file 2: Figure S3). Moreover, when a significant distribution (p < 0.01) of tiles localize to “undefined” space, outliers can be efficiently labeled as “undefined” without the need for empirical cutoff scores (Fig. 4a-b). Collectively, this novel approach offers a balance of visually intuitive, objective and “graded” performance metrics for routine histomorphologic analysis.

### t-SNE-based classification performance

**Fig. 5:**
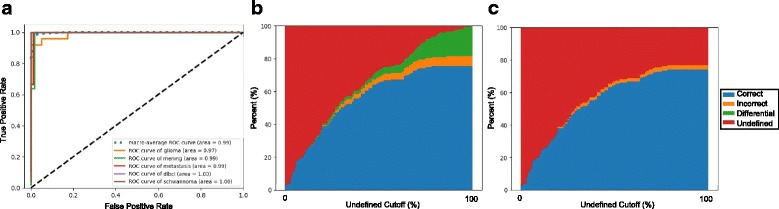
Composite approaches to WSI classification. **a** ROC curves generated using a hybrid prediction score comprised of a blend of the percentage of tile distribution and probability scores across the different classes. This approach achieved an even higher AUC. **b** Relationship of accuracy (y-axis) of this hybrid classifier at different “undefined” score cutoffs of the t-SNE component (% of tiles in undefined space). **c** Relationship of accuracy of another more conservative scoring approach where only concordant cases are classified at different “undefined” score cutoffs. This approach further reduces the overall error rate with a minimal change to the number of classified cases compared to t-SNE alone

To test the performance of this alternative approach, we subjected the same 180 cases through a similar workflow but substituted probability-based predictions with our t-SNE-driven metrics (Fig. 2a). For each test slide, however, this approach, in addition to rendering diagnoses, also had the flexibility to signal uncertainty (“undefined” or “differential diagnosis”). Similar to the probability scores, for cases where a single diagnosis could be statistically reached (based on *χ*^2^ testing), the distribution of tiles among the different tissue classes was used to generate a mROC curve. The AUC, similar to using probability scores, was high (AUC = 0.99, Fig. 4c). Perhaps more importantly, this conservative and statistically-driven metric disproportionally reduced errors (mostly Type B & C) without the need for *post-hoc* tuning of cut-off scores [t-SNE: 4% error, 68% correct vs. probability distribution scoring: 16% error, 84% correct) (Fig. 4d). Error reduction was largely attributed to a strong buffer against “Type C errors” (untrained classes) which were enriched with “undefined” lesional tiles on t-SNE plots (Fig. 4a-b). Intriguingly, even though these two different approaches stemmed from the same trained CNN, the slightly different approaches to classification led to non-overlapping errors. In a *post-hoc* analysis, we found that composite approaches, where both tests were in agreement, could be leveraged to further reduce error rates (combined: 2% error, 67% correct) and/or improve overall classification rates (Hybrid, mROC: 1.00, Fig. 5). Minor customized optimizations of this generalizable classification approach offers new quantitative visualization tools for large-scale morphologic analysis and quality control in practical real-world settings.

**Fig. 6:**
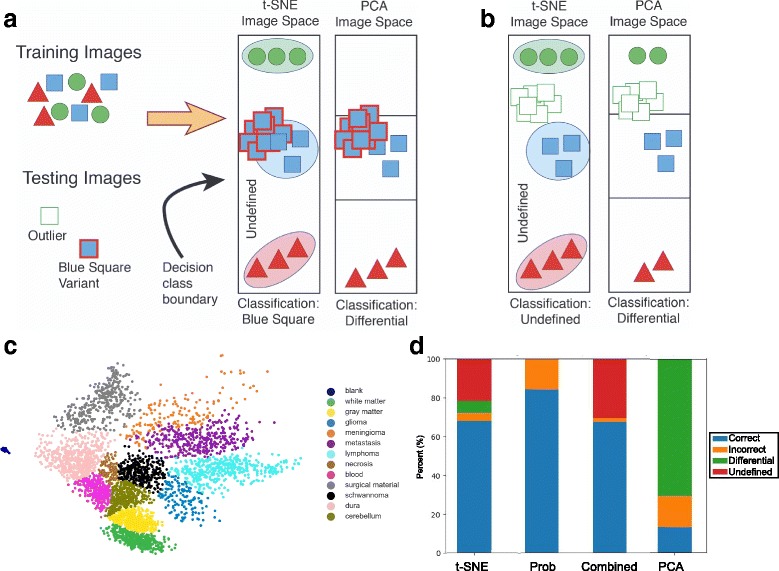
Alternative dimensionality reduction approaches. **a**-**b** Hypothetical cartoon depicting advantages of the t-SNE plot as a “graded” and complementary classification tool. **a** Representative image spaces within a CNN as organized by different dimensionality reduction techniques. Unlike linear representations (e.g. PCA) with discrete and adjacent decision borders, the exaggerated separation of classes on the t-SNE plot provides more distinct categorical classes for testing. This key difference has significant advantages for evaluating the distribution of test tiles amongst previously trained classes. As shown, this could allow more effective handling of variants of already trained cases (“blue square variant”, Panel a) and true “undefined” classes (green square, Panel b). **c**-**d** For comparison, we show the PCA depiction of our trained CNN. Although similar in the overall arrangement of classes, there is notably little separation between tissue types. This difference leads to the vast majority (71%) of cases being classified as “differential” and a substantial decrease in performance (13% correct and 16.2% errors compared to both t-SNE and probability distribution scoring (Prob). Correct cases of the “combined” analysis represents those where both the t-SNE and Prob score were in agreement. The undefined class in the “combined” analysis represents cases with classification discordance between the two methods

As a final analysis, we compared the performance of our t-SNE approach to principal component analysis (PCA), the most common linear dimensionality reduction techniques (Fig. 6). Similar to t-SNE, PCA can depict high-dimensional data stored in CNNs on a two-dimensional plane. We hypothesized, however, that the lack of exaggerated spacing between classes would preclude effective discretization of classification boundaries as we observed on our t-SNE plot (Fig. 6a-b). This hypothesis was indeed correct (Fig. 6c). The proximity of the classes and lack of an “undefined” category to buffer challenging cases led to a substantial reduction in the performance of how slight variants of already trained (and untrained) classes were handled (Fig. 6c-d).

## Discussion

Although a well-established visualization tool for high dimensional data [6, 11], utilization of t-SNE for interpreting histomorphologic machine learning is scarce. We demonstrate novel and generalizable applications of t-SNE as a tool to provide informative insight of how histology-based learning is stored within neural networks. Unique to our study, we show how this organizational structure can be used to generate alternative classification outputs that can be visualized and help substantially reduce errors. Notably, these benefits were afforded without the need for additional CNN training or significant adjustments to standard CNN-based histologic workflows. Our t-SNE-based classification approach showed very similar classification performance to more traditional and less transparent probability-based distribution scores. Although slightly more conservative in classification, unclassified cases were substantially enriched for outliers. Together, the improved transparency of visual outputs and lower tolerance for unfamiliar features could provide value for industries (e.g. healthcare, autonomous driving) where the benefits of error/outlier detection outweigh other performance parameters (e.g. % correct on first pass). Overall, even without *post-hoc* tuning, we were able to substantially reduce erroneously classified cases (both Type B/C errors) (4% vs. 16%). The non-overlapping errors of the different approaches could allow development of additional composite metrics that further reduce errors without the need for cutoff optimization. The error rates achieved by our tool are in line with baseline reports of inter-pathologist discrepancies. We thus believe this composite approach, when coupled with final expert human review, could be an especially valuable companion in improving efficiency and quality control at remote under-serviced centers. “Undefined” classifications could also be utilized to prioritize challenging cases for extended subspecialist-, immunohistochemical-, and molecular-based diagnostic interrogation.

There are some important considerations to highlight regarding our approach. The vast majority of residual misclassified cases by the conservative t-SNE approach represented tumor subtypes (e.g. atypical meningiomas, small cell glioblastomas) that were not found in the training set (“Type B errors”). These cases often had subtle features that resembled the erroneously chosen class. Clinically, these more challenging cases often prompt confirmatory immunostaining, molecular studies, and/or supportive clinical history to confidently resolve. We believe that some of these “Type B errors” could be further reduced, or appropriately labeled as “differential diagnosis”, with additional training examples. It was beyond the scope of the study to optimize performance and reduce errors by tuning these diverse variables or adding additional, relatively rare classes. Instead, we aimed to develop an alternate visual and statistically-driven classification approach that is applicable to scenarios that extend outside of the CNN’s original training environment. Moreover, although our test set included rare subtypes of tumors never seen by our classifier, it is likely still an under-representation of the true diversity and challenges seen in more “practical” real-world settings. Firstly, assembly of the presented test set spanned a relatively short timeframe (3–4 months) and was limited to cerebral lesions in which pathologist reached consensus. Long-term application of such generalized decision support tools in pathology are likely to encounter many additional temporally dependent, technically-derived artefacts and outliers. Moreover, other rare non-neoplastic and extra-cranial neuropathologies not integrated into the classifier routinely arise. This diversity makes comprehensive CNN training and empirical cutoff selection an extremely challenging and perhaps futile task in the short term. Our t-SNE classification approach, that we show is extremely resilient to unanticipated outlier cases, provides a more immediate solution to many histology-based classification tasks.

While classification of images using neural networks has been extremely successful, there are important caveats to be mindful of. Unlike controlled competitions, like the ImageNet challenge, in which there is a fixed and pre-defined number of classes, pathologists routinely encounter extremely rare cases in which they may see once or twice in their whole career. Moreover, disease morphologies and classification of more common lesions could dramatically change with the introduction of new surgical and diagnostic procedures, medical therapy and early screening. The lack of training images for these rare and changing morphologies may challenge use of CNNs in clinical practice. Similarly, in the ImageNet challenge, having an object listed in the “top 5” by probability score is considered “correct”. In diagnostic pathology however, only the favored diagnosis often adds value and is actionable by the clinician. Error rates (~ 20–25% errors) of CNNs in ImageNet challenges when only the class with the highest probability (“Top-1”) is used to calculate performance is still far from the level needed for clinical use. Current state of the art pathology workflows substantially reduce this error rate through the use of molecular testing and additional clinical information. It is therefore likely that, at least in the short term, CNN pathology workflows should be designed to serve as decision support tools that improve the throughput of pathologists rather than replacing them. We believe our novel classification is well suited from this role.

Lastly, we believe our visual and quantitative approach for classification and anomaly detection will likely benefit from further optimization of variables. These include image patch size, number of trained classes, understanding the degree of pattern differences between classes, CNN architectures/training approaches and different threshold cutoffs within the workflow. We highlight some of these considerations within our manuscript (See Additional file 2: Figure S4). In our experience, however, the principles and error reduction properties we outlined in our classification approach are largely conserved. Our approach thus serves as a strong and generalizable framework that can be easily be adapted for anomaly detection in other disciplines of histopathology and related fields.

## Conclusion

Currently, most deep learning solutions in pathology rely on probability distribution score outputs. We introduce an alternative classification approach that provides highly visual, intuitive, and graded classification metrics. We highlight how our novel approach helps substantially reduce errors by efficiently labeling outliers without the need for extensive *post-hoc* optimization of cutoff values. This simple approach could therefore accelerate adoption of CNN-based technologies into pathology and other sectors where high error rates compromise efficiency, cost-effectiveness, and safety. Our workflow can be theoretically applied to multiple tissue and disease classes across the diverse pathology subspecialties. We thus present a highly generalizable approach to quantitatively visualize AI-based decision making and anomaly detection in practical real-world settings.

## Additional files

Additional file 1 **Table S1.** Distribution of tissue types and images used for the development of the CNN training dataset. (PDF 153 kb)

Additional file 2 **Figures S1-S4.** Additional data figures cited throughout the manuscript. (PDF 2768 kb)

## Abbreviations

AI: Artificial intelligence
AUC: Areas under the ROC curve
CAM: Class activation map
CNN: Convolutional neural network
GFAP: Glial fibrillary acidic protein
GPU: Graphical processing unit
H&E: Hematoxylin and Eosin
IDH: Isocitrate dehydrogenase
PCA: Principal component analysis
REB: Research ethics board
ROC: Receiver operator characteristic
t-SNE: t-distributed stochastic neighbor embedding
WHO: World Health Organization
WSI: Whole slide image
