# Supplementary material for: Visualizing histopathologic deep learning classification and anomaly detection using nonlinear feature space dimensionality reduction
Source: BMC Bioinformatics. 2018 May 16;19:173. doi: 10.1186/s12859-018-2184-4 (PMC5956828; doi:10.1186/s12859-018-2184-4)
Supplement: Supplementary file 2 — Table S1. Distribution of tissue types and images used for the development of the CNN training dataset. (PDF 153 kb) [file 12859_2018_2184_MOESM1_ESM.pdf]

Supplementary Table 1 | Distribution of tissue types and images used for initial CNN training. Images numbers represent 1024 x 1024 pixel images.

Class	Unique Cases	Unique Images (Total)	Unique images selected for training
**Normal Tissue Types**			
White Matter	15	4,933	3,455
Gray Matter	17	7,431	6,244
Cerebellum	4	7,169	2,554
Dura	6	910	638
**Tumor Types**			
Glioma	41<br>- 20 Glioblastoma, IDH wildtype, WHO grade IV<br>- 11 Astrocytomas, IDH mutant, WHO grade II-IV<br>- 10 Oligodendroglioma, IDH mutant, 1p19q co-deleted	18,948<br>7,169<br>6,391<br>5,388	7,000
Meningioma	28<br>- 9 Meningothelial, fibrous, transitional Meningioma subtypes, WHO grade I<br>- 9 Psammomatous Meningioma, WHO grade I<br>- 7 Microcystic/Angiomatous, WHO grade I<br>- 3 Secretory Meningioma, WHO grade I	15,504<br>7,380<br>1,529<br>4,855<br>1,740	7,000
Metastasis	11<br>Subtype: Adenocarcinoma (breast, lung, esophagus, colon)	4,043	2,831
Schwannoma	6 (conventional)	3,760	2,632
Lymphoma	5 (Diffuse Large B-Cell Lymphoma)	1,939	1,359
**Other tissue types**			
Blood	23	5,102	3,572
Surgical Material	7	368	315
Necrosis	13	4,164	2,749
Blank	10	10,232	7,182
**Total:** 13 Class	122 (unique cases)	84,503	47,531
