# Supplementary material for: Visualizing histopathologic deep learning classification and anomaly detection using nonlinear feature space dimensionality reduction
Source: BMC Bioinformatics. 2018 May 16;19:173. doi: 10.1186/s12859-018-2184-4 (PMC5956828; doi:10.1186/s12859-018-2184-4)
Supplement: Supplementary file 3 — Figures S1-S4. Additional data figures cited throughout the manuscript. (PDF 2768 kb) [file 12859_2018_2184_MOESM2_ESM.pdf]

Supplementary Figure 1 *Faust et al.*

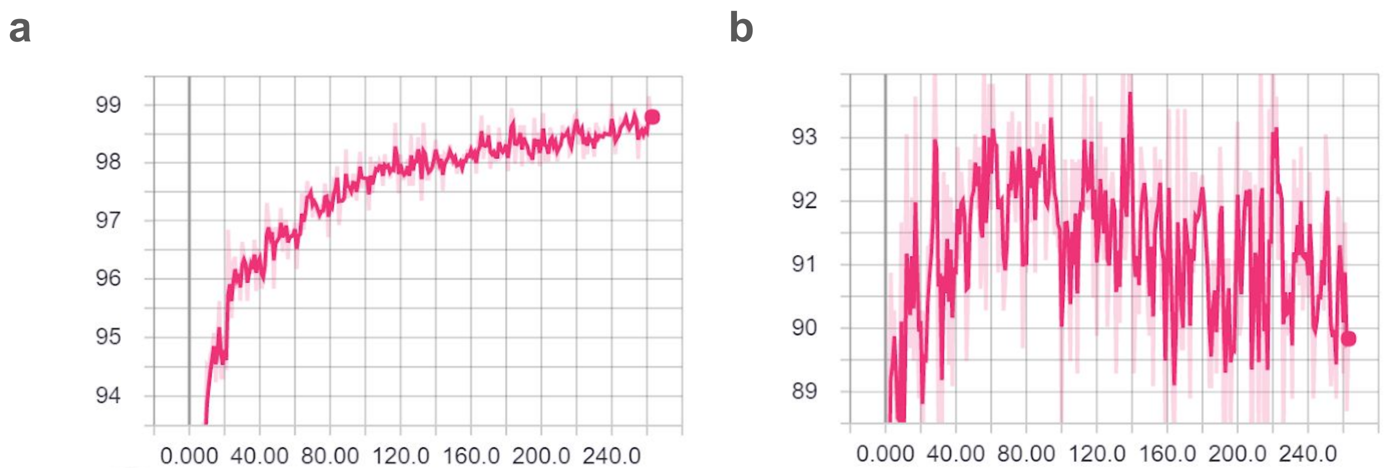

Supplementary Figure 1 | Line graphs plotting training (a) and validation (b) accuracy (%, y-axis) after each epoch (x-axis). The best performing model reached a validation accuracy of 94.8%.

Supplementary Figure 2

Faust et al.

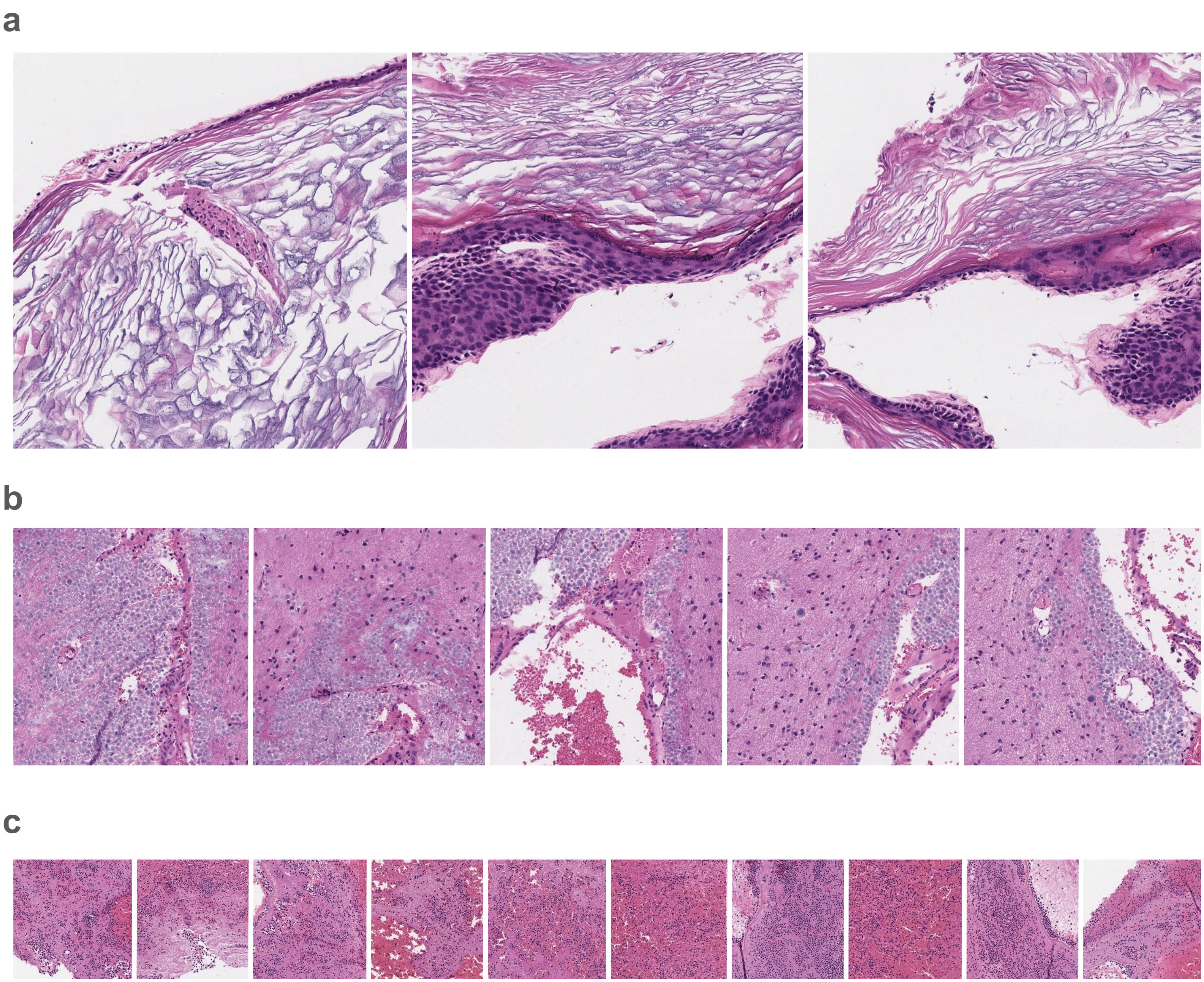

Supplementary Figure 2 | Sample output files for WSIs that did not reach initial >15 tile cutoff for classification . Following tiling of an entire WSI, each 1024 x 1024 pixel image patch is classified using a modified 9-class lesional CNN classifier (**Figure 2a**). Only tiles with a probability distribution score of >85% "lesional class" are used for classification. If less than 15 tiles reach this cutoff, classification is not performed. Instead the few "lesional" tiles are assembled into a JPG image (as shown here) for convenient human review. Panels above show JPG output strips from a WSI with a true label of epidermoid cyst (n = 3 "lesional tiles") (**a**), focal cortical dysplasia with abundant corpora amylacea (n=5) (**b**), and a hematoma (n= 10) (**c**)**.** All tiles shown have a length of 516 micrometers.

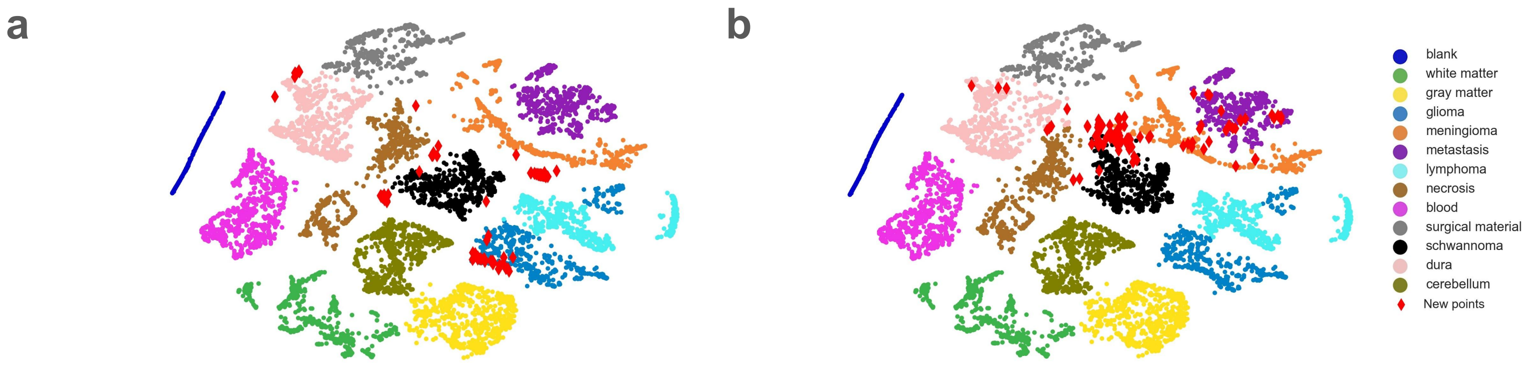

Interpretation: Differential diagnosis (p_value: 8.3265E-02)
g_glioma, q_schwannoma

Breakdown of TSNE class points within the slide
Undefined Anomaly: 48
g_glioma: 30
q_schwannoma: 18
r_dura: 3
h_mening: 1

Ground Truth: Glioma

Interpretation: Differential diagnosis (p_value: 6.4565E-01)
k_metastasis, q_schwannoma, h_mening

Breakdown of TSNE class points within the slide
Undefined Anomaly: 48
k_metastasis: 18
h_mening: 17
q_schwannoma: 13
r_dura: 4

Ground Truth: Metastasis

Supplementary Figure 3 | Sample output files for WSIs that could not reach a diagnosis following statistical testing ("differential diagnosis"). t-SNE tile coordinates from each test WSI are used to generate a contingency table for statistical testing. A serial chi-square testing approach assess if tile distributions are significantly different between categories. Each time a significantly skewed distribution is reached, the class with the fewest tiles is discarded and the remaining classes are again assessed for goodness-of-fit. This either yields a signal diagnosis (see **Figure 2** for examples) or a "differential diagnosis" as shown here. The classes that did not significantly differ in distribution are listed along with the non-significant *p*-value. Although the correct diagnosis was often the most populated class and/or among the list of remaining diagnosis, these cases were not utilized for performance testing (correct vs. incorrect). Panels above show a glioma (**a**) and metastasis (**b**) that did not reach significance for a single class.

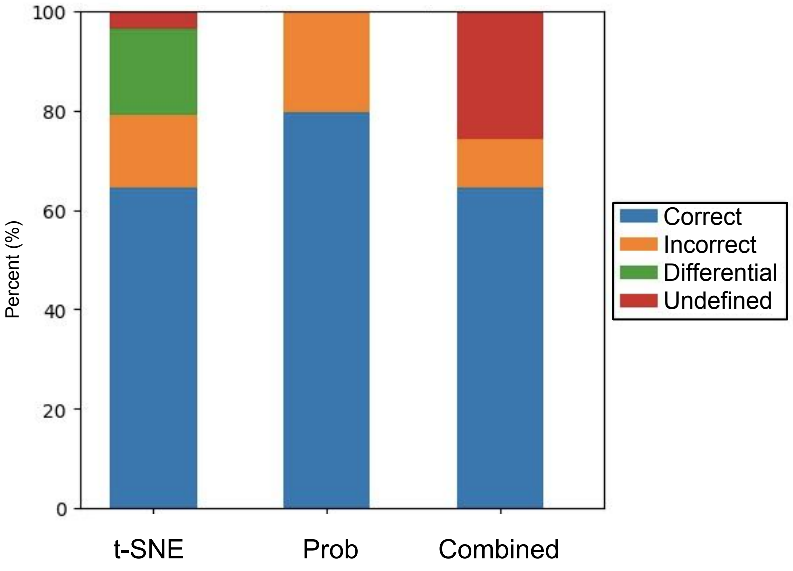

Supplementary Figure 4 | Performance metrics for our classification workflow at different classification thresholds. Bar graphs show the number of cases classified as “correct”, “incorrect”, “differential diagnosis” or as “undefined” when >5 tiles could be extracted from a test image. Although this led to more images being classified, most represented WSI with no pathological diagnosis (examples Supplementary Figure 2). This also led to an increased error rate in all the tested approaches due to misclassification of poorly sampled non-diagnostic tissue (e.g. hemorrhage, normal brain tissue). t-SNE error rate rose from 4% to 15%, while “Prob” and combined error rate error rate rose to 20% and 10% respectively.
